# 1,3-Pentadiene-Assistant Living Anionic Terpolymerization: Composition Impact on Kinetics and Microstructure Sequence Primary Analysis

**DOI:** 10.3390/polym15092191

**Published:** 2023-05-05

**Authors:** Qiaoqiao Xiong, Yawen Fu, Jundong Xu, Zhuowei Gu, Chengjun Peng, Haoyun Tan, Qiqi Dai, Yujie Cao, Fengli Xie, An Li, Wenjun Yi, Lijun Li, Kun Liu

**Affiliations:** 1 Province Key Laboratory for Fine Petrochemical Catalysis and Separation, College of Chemistry and Chemical Engineering, Hunan Institute of Science and Technology, Yueyang 414006, China; xiongqiaoqiao1128@163.com (Q.X.); woxiangjiwode@126.com (Y.F.); xujdai@126.com (J.X.); guzhuowei1202@126.com (Z.G.); pcj13187310809@163.com (C.P.); thy1849624144@163.com (H.T.); dqq1390811831@163.com (Q.D.); 15273031626@163.com (Y.C.); xiefengli2023@163.com (F.X.); anleechn@126.com (A.L.); 2 College of Materials Science and Engineering, Changsha University of Science & Technology, Changsha 410082, China

**Keywords:** poly(1,3-pentadiene-*co*-syrene-*co*-1,1-diphenylethylene) resins, living anionic technology, unique alternating strategy, kinetic analysis, “bond-forming initiation” theory

## Abstract

The combination of a living anionic technology and a unique alternating strategy provided an exciting opportunity to prepare novel and well-defined poly(1,3-pentadiene-*co*-syrene-*co*-1,1-diphenylethylene) resins consisting of three alternating sequences of modules (**A/B/C** zwitterions). “A” being Styrene (St)/1,3-pentadiene (PD), “B” being diphenylethylene (DPE)/PD, “A” being DPE/St, respectively, A wide composition range of new polyolefin resins, i.e., poly (**A**-*co*-**B**), poly (**A**-*co*-**C**), and poly (**B**-*co*-**C**), with controlled molecular weight and very narrow molecular weight and composition distributions have been prepared by a one-pot living characteristic method. In the section of kinetic analysis, the terpolymer yields and kinetic parameters were strongly dependent on the feed/comonomer ratio as well as the content of the alternating structure. The competition copolymerization behaviors of **A/B**, **B/C**, and **A/C** were studied in detail in this work. By contrast, the microstructure and the thermal property of the resulting terpolymer were investigated via Nuclear magnetic resonance (NMR) and Differential scanning calorimetry (DSC) analysis. The results of 1H NMR tracking the change of [Aromatic ring]/[C=C] value indicated the distinctive copolymer-ization behavior of the selective “alternating-modules”. The glass transition temperature (*T*_g_) was very sensitive to the terpolymer composition. By contrast to poly(**A**-*ran*-**B**) with only one obvious *T*_g_, there were two *T*_g_s in the **A/C** and **B/C** copolymerization cases. Moreover, the desirable high *T*_g_ ~ 140 °C resin was limited to the terpolymers with up to 50 mol % DPE. Finally, the “**ABC-X**” mechanism was proposed to interpret the unique terpolymerization behavior, which belongs to the classical “bond-forming initiation” theory.

## 1. Introduction

Living sequence-controlled polymerizations from a mixed unsaturated hydrocarbon feedstock offer the possibility of producing polyolefin materials with desired and unexpected properties [1,2,3,4,5,6]. Undoubtedly, the anionic polymerization has been highly efficient to produce these polymers; however, it suffers from poor monomer diversity and selectivity. Styrene (St), butadiene (BD), and isoprene (IP) are considered to be the most important but limited monomers for synthetic rubbers or Thermoplastic Elastomers (TPEs), well-known as styrene butadiene styrene block copolymer (SBS) and Styrene isoprene styrene block copolymer (SIS) as well as their hydrogenated products [7,8,9,10]. In fact, 1,3-pentadiene (PD), so far clearly underestimated 1,3-diene, is also a cheap and abundant feedstock from the convenient steam cracking of naphtha [11]. As for this unique monomer, the cationic polymerization offers a simple but uncontrolled strategy for synthesizing C5 petroleum resin, which is widely used as an adhesive [12,13]. On the contrary, the synthesis of diene-based polymers via living anionic copolymerization offers the possibility of providing a wide variety of materials, including multi-block polymers, random rubbers, alternative polymers, end-functionalized polymers, and other complex polymer architectures [14,15,16].

The control of the monomer sequences at the molecular level is still one of the “holy grails” in polymer science [17,18,19]. Among the various monomers reported to date, PD is a suitable candidate for anionic copolymerization to allow the synthesis of polymers with much more periodic sequences. In contrast to BD or IP, the anionic copolymerization of PD with St/DPE can successfully generate strictly alternating copolymers due to the selective crossover propagation reaction [20,21]. Previously, pioneering works only demonstrated the possibility of synthesizing alternating copolymers by carbanionic copolymerization of bulky comonomers (such as St/DPE [22], 2,3-dimethyl-1,3-butadiene (DMBD)/DPE [23], St/1,3-cyclohexadiene (CHD)) [24], either of which cannot or hardly undergo self-propagation but cross-propagation to form alternating sequences. In fact, still tremendous works have been performed in this decade for obtaining chain-end and chain-middle functionality petroleum-based polymers by the classical “DPE chemistry” strategy [25]. To the best of our knowledge, fewer studies are dealing with the alternating/statistical/block/tapper/random copolymerization or terpolymerization of PD with common monomers in an anionic family.

**Scheme 1:**
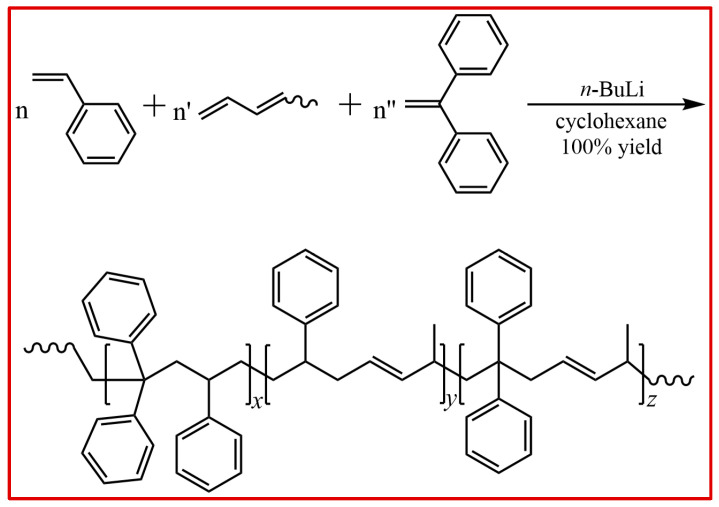
Living anionic terpolymerization of 1,3-pentadiene with styrene and 1,1-diphenylethylene, producing a quantitative terpolymer containing three alternating structures.

The aim of this study was to investigate the terpolymerization behavior of St and DPE with PD. Poly (DPE-*alt*-PD) shows a glass transition at 125 °C but behaves as a very brittle material at high molecular weights. In contrast, poly (St-*alt*-PD) is considered to be a soft polymer, especially when hydrogenated. The combination of both alternative copolymers in a so-called terpolymer offers the possibility to overcome the unfavorable properties and to obtain a polymeric material with tunable thermal and mechanical properties. This route provides access to well-defined terpolymers with tunable compositions and molecular weights (Scheme 1). Furthermore, when the feed ratio is appropriate, both comonomers can form three alternative units (**A/B/C**) in the terpolymerization process when the feed ratio is appropriate, indicating that the two alternating sequences are statistically incorporated. This result opened a promising route to novel terpolymers exhibiting mixed-glass transition temperatures.

In this work, the reaction kinetics of the terpolymerization reaction was studied in detail to obtain a better insight into the interesting polymerization behavior. In addition, the terpolymer synthesized via the living/controlled approach was more closely investigated in terms of the constant [Aromatic ring]/[C=C] compositions and monomer conversions to find out if the difference in the sequence distribution is reflected in any change in the feedstock ratio. Differential scanning calorimetry (DSC) was used for glass transition analysis.

## 2. Results and Analysis

**Scheme 2:**
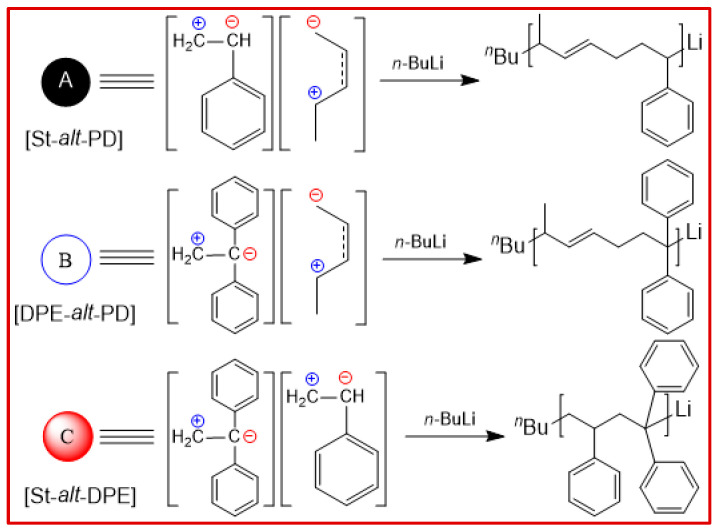
Three alternating sequences of **A/B/C** composed of two comonomers in this work.

Our previous work reported the living alternating anionic copolymerization of PD with St or DPE using various solvents, such as cyclohexane or toluene. We also investigated the kinetics of the alternating binary polymerization of PD in details by NMR spectroscopy and the gravity method. It was shown that both comonomers have identical propagation rates when both of the selected monomers are mixed in the reaction solution. This is interpreted by the zwitterionic state theory that M_1_ and M_2_ interacted as a 1:1 complex prior to initiation by carbanionic species. As shown in Scheme 2, here, we select the St-PD-DPE terpolymerization system, in which the terpolymerization proceeded mainly by “**ABC-X**” mechanisms in the state of near-binary copolymerization of **A** (St¦PD) and **B** (DPE¦PD) or **C** (St¦DPE) intermediates with residual monomer **X** (i.e., St, DPE, PD). Conceivably, living and controlled polymerization has an overwhelming advantage in designing and creating novel sequences based on the feed ratio (**A/B**, **B/C** and **A/C**) as well as the differences in the reactivity ratio (*r***_A_**, *r***_B_**, and *r***_C_** as well as *r*_St_, *r*_PD_). Thus, the “gradient”, block, random, and alternating copolymers containing strictly **A/B/C** alternation units in the well-defined polymer chain can be generated by living/controlled polymerization. The copolymerization behavior and the key composition effect on polymer yield, i.e., the random incorporation of the selected alternating sequences into the polymer backbone during the copolymerization process, are discussed as follows.

**Figure 1:**
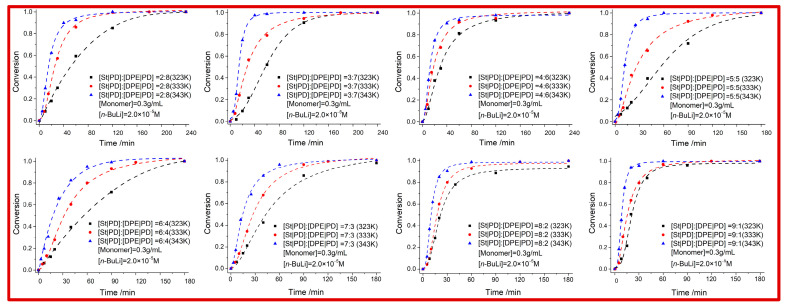
The kinetic curves of conversion with time at different [**A**]/[**B**] ratios.

**Terpolymerization kinetics analysis**. In the case of **A/B** = 1/1 copolymerization (i.e., St/PD/DPE = 1/2/1), equimolar **A** and **B** alternating units can be successfully inserted into the main chain, and the “one-pot” copolymerizations were completed with quantitative conversions (>99% in Table 1). The resulting terpolymer showed rather narrow molecular weight distribution and controlled molecular weight (*Ɖ* = 1.08, *M*_n_ = 25.5 kDa, Table 1). Intrigued by this result, the copolymerization experiments with different **A/B** ratios (from 2/8 to 8/2) were immediately carried out in cyclohexane at 50–70 °C. The given 100% yields revealed that the composition of the terpolymer could be adjusted simply by changing the corresponding ternary feed ratios, although the incorporation rate of **A** was slightly faster than that of **B** due to its relatively high alter-propagation rate under the similar experimental condition [26]. In fact, we could easily obtain a family of **A**-*co*-**B** polymers with designed **A/B** alter-sequence contents to satisfy the demand of the versatile material. To our delight, all the GPC traces of the isolated terpolymers were monomodal (*M*_n_s = 21.9–31.9 kDa, Table 1), and the molecular weight distributions remained in a much narrower range (*Ɖ*_M_s = 1.07–1.12, Table 1). As shown in Figure 1, the copolymerization rate decreased slightly with the increasing of the **B** content in the mixed monomers. Considering these typical anionic non-double S curves, it can be conceived that both **A** and **B** can not only undergo homo-propagation but also undergo cross-propagation to extend the chain. Moreover, the crossover rate from **A** to **B** is much faster than that from **B** to **A**; otherwise, the P**A**-*b*-P**B** or at least the tapered block copolymer would be synthesized, and the double-S cure would be displayed. Of course, the sequence distribution would be verified through the aforementioned NMR analysis.

**Figure 2:**
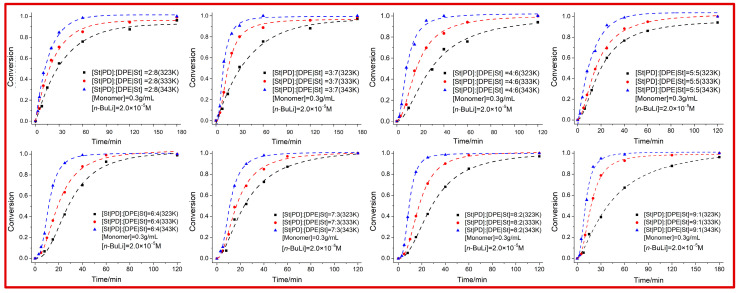
The kinetic curves of conversion with time at different [**A**]/[**C**] ratios.

For the equimolar copolymerization of **A** and **C**, the well-defined poly(**A**-*co*-**C**) with very narrow weight distribution (*Ɖ* = 1.09) was also successfully synthesized as expected. As shown in Figure 2, initiated by *n*-BuLi, all comonomers can be completely consumed within 1 h at 70 °C. Theoretically, DPE as a comonomer hardly can be completely converted to a copolymer due to the homopolymerization probability of the non-DPE comonomer. When the styrene is consumed, the propagation reaction will end as a DPE-capped living species, although excess DPE will be added to the living ~S-Li ends. At this point, it is impossible to obtain the designed and precise composition of the copolymer, which is in accordance with the monomer feed ratio. However, the yield was still 100% even when the feed ratio of *f***_C_** exceeded 80%, showing that the homopolymerization reactivity of St was completely depressed with the addition of a PD monomer. These results showed no difference with our previously reported work [27]. Therefore, the DPE comonomer had enough time to polymerize with St to form the strictly alternating **C** sequence before the insertion of the excess St monomers into the **AA** structure, and thus, the final composition will inevitably be equal to the molar feed ratio. Especially in the case of **A/C** copolymerization, it seemed that the copolymerization rates had little relationship with the feed ratios. To prove the living characteristic of the end species, an amount of PD or St monomer was added to the red solution to restart the DPE insertion. Then, the terpolymer with 100% DPE incorporation can also be skillfully produced. Of course, we cannot illustrate the precision copolymerization model without further sequence information given for the time being.

**Figure 3:**
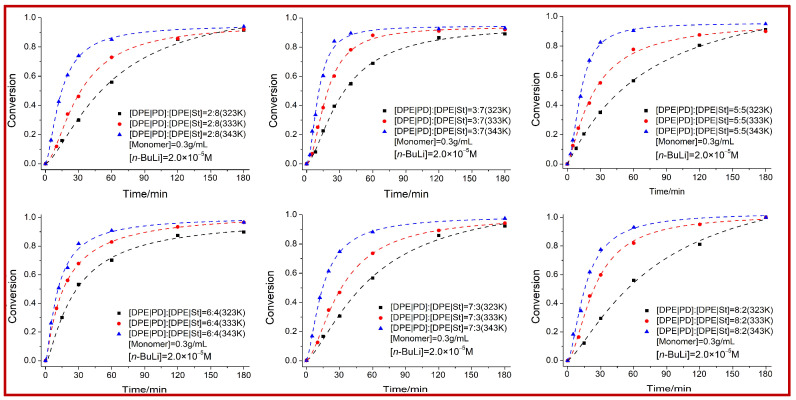
The kinetic curves of conversion with time at different [**B**]/[**C**] ratios.

**Figure 4:**
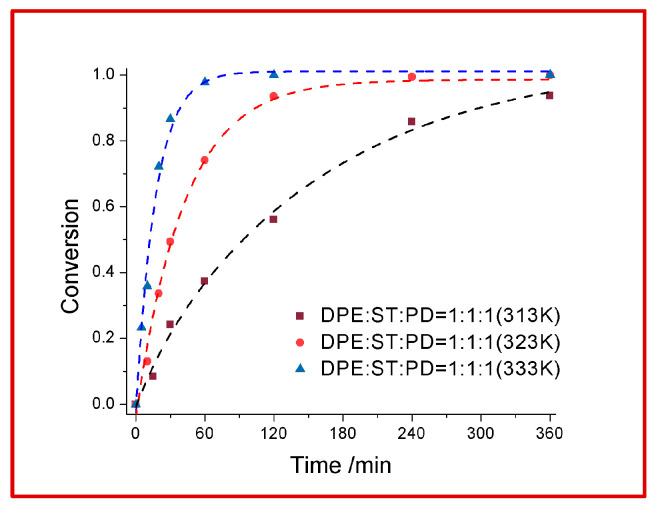
The kinetic curves of conversion with time at DPE/St/PD = 1/1/1.

The DPE/St/PD = 2/1/1 (i.e., **B**/**C** = 1/1) terpolymerization was carried out with *n*-BuLi under various conditions as shown in Table 1. Contrary to the **AB** and **AC** systems, the comonomer feed ratio in each experiment has a significant effect on the conversion as well as the composition of the copolymer; although, it has little influence on the well-controlled characteristic of the terpolymerization (*Ɖ* = 1.06–1.13, Table 1). Specifically, as shown in Figure 3, high conversion (>99%) required a relatively high feed ratio of **C** (>70% molar ratio), and the copolymerization rate increased slightly with the increasing content of the **C** comonomer content. At *f***_C_** > 0.5, the 5–10% excess of DPE was impossible to copolymerize with St via a crossover reaction to form alternating sequence units. The SS block or PDPD block moieties were inevitably formed owing to the mismatched reactivity ratio (*r*_St_ >> 0) and the stability of the zwitterionic intermediate **B**, i.e., the low concentration of PD has priority to interact with equimolar DPE and thus has limited inhibition of SS homo-propagation reactivity. To explore the competitive behavior of St and PD when copolymerized with DPE, we investigated the DPE/St/PD = 1/1/1 terpolymerization kinetics as shown in Figure 4. Considering the much lower homopolymerization rate of PD than St, DPE has high priority to react with PD to insert this alternating sequence into the polymer chain. Thus, it can be considered as nearly binary copolymerization of St and **B**.

**Figure 5:**
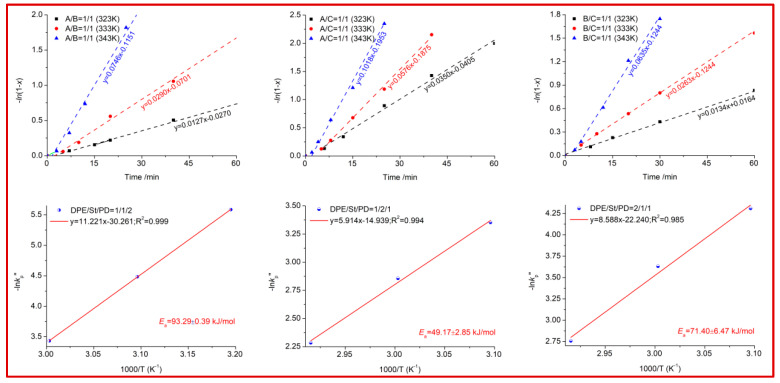
The kinetic *E*_a_ parameters of DPE/St/PD terpolymerizations with different feed ratios.

Kinetic parameters were calculated from the conversion versus time data in the range of 50–70 °C, and the results are shown in Figure 5. All the ternary copolymerization experiments strictly followed the first-order reactions {*R*_p_ = *k*[*^n^*BuLi]*^x^*[**A**]*^a^*[**B**]*^b^*[**C**]*^c^*, (*a + b + c* = 1)}, regardless of the feed ratios. Furthermore, we found that the copolymerization *E*_a_ values had a direct relationship with the feed ratio. In our previous work, the *E*_a_ (*ln*A) values of **A** homopolymerization (i.e., St/PD alternating system) and **B** homopolymerization (i.e., DPE/PD alternating system) were 85.15 ± 2.43 kJ/mol (28.13 ± 0.91) and 21.97 ± 3.35 kJ/mol (4.78 ± 1.29), respectively [26]. Herein, the determined kinetic parameters showed that the *E*_a_ value dramatically decreased with the increasing content of the **B** and **C** “monomer”, but the unparalleled copolymerization rate may be attributed to the steric constraints of phenyl groups in **B/C** structures. In particular, the *E*_a_ value calculated at a 1/1 feed ratio are shown in Figure 5: **A/B** > **B/C** > **A/C**. The PD-rich terpolymerization rate was much more insensitive to temperature than the St-rich system, while the DPE-rich system has similar polymerization activity, indicating that the steric hindrance effect should be another key factor. The logarithmic conversion data, −*ln*(1−*x*) plotted against *time*, gave straight lines. We can estimate the apparent *k*_p_” values from the slopes in the case of the first-order terpolymerization. It seemed that the kinetic order of the terpolymerization was independent of the monomer ratios. “Random” or at least “statistical” rather than “block” copolymerization of binary “alternating” sequences was possible to interpret the kinetic mechanism. No significant difference of homopolymerization activity between these “alter-modules” (**A** and **B** or **C**) allowed to simplify this copolymerization model.

**Figure 6:**
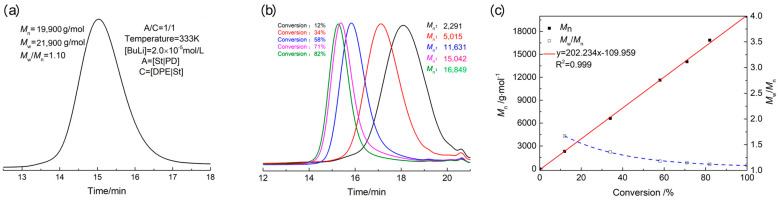
(**a**) GPC curves of poly (**A/C** = 1/1) prepared with *n*-BuLi in cyclohexane (*M*_n_ = 19.9 kg·mol^−1^, *Đ* = 1.10); (**b**) *M*_n_s and *Đ*_M_s for the obtained poly (**A**-*co*-**C**) alternating polymers; and (**c**) molecular weight distributions for the alternating copolymerization of **A** and **C** using *n*-BuLi as initiator at 70 °C.

As shown in Figure 6, the typical St-rich terpolymer had unimodal MWDs, and the *Ɖ*_M_s values decreased dramatically with increasing conversions. Notably, the *M*_n_ values of the terpolymers increased linearly as the polymerization progressed, indicating that long-lived species were constantly generated during polymerization. The *M*_n_ values determined by polystyrene calibrated GPC were relatively consistent with the theoretical values. Overall, the results of the terpolymerization reactions have perfectly well-controlled and living characteristics. The PD-rich and DPE-rich terpolymerizations were also found to have the same polymerization characteristics.

Interestingly, the proportions of the C=C bond of the diene and the phenyl-ring of the aromatic monomer in the terpolymers were found to be identical to those of the feed components. This quasi-quantitative incorporation of both monomers can be explained by taking into account their close copolymerization activities (**A/B/C**) but can appear as surprising when considering the much lower reactivity of PD compared to St. In fact, the DPE or PD can be considered as non-homopolymerized monomer in the carbanionic initiating system under low temperature (*r.t.*). The steric hindrance generated by the DPE, and its strong inductive effect compared to that of St, may be responsible for this unique behavior. This terpolymerization is an example of termonomer-induced alternating copolymerization (TIAC), which associates monomers that do not copolymerize or have a much lower homopolymerization rate (DPE or PD), and also a termonomer (St) that exhibits a good crossover propagation to both of them. Hence, the polymerization reactions between DPE, St, and PD were monitored by NMR spectroscopy to understand how these two alternating sequences are arranged. The reaction was carried out under similar conditions as for the poly[(DPE-*alt*-St)-*co*-(DPE-*alt*-PD)] and poly[(DPE-*alt*-St)-*co*-(St-*alt*-PD)] and poly[(St-*alt*-PD)-*co*-(DPE-*alt*-PD)] terpolymers, respectively. Samples were taken from the reaction media at regular time intervals to monitor the evolution of the incorporation of [C=C] and [Aromatic ring] incorporation into the polymer by NMR spectroscopy versus the conversion of monomer.

**Microstructure and sequence primary analysis.** All poly-(DPE-*ter*-PD-*ter*-St) terpolymers with different molar ratios (1/1/2, 1/2/1, and 2/1/1) were investigated by ^1^H NMR (Figures S4–S7) and GPC analysis. Similar molecular weights and molecular weight distributions of the products were observed, indicating that the addition of the third comonomer did not significantly alter the polymerization characteristics (similar propagation rate, no additional termination reaction). The high molecular weight and narrow molecular weight distribution are indicative of an ideal living/controlled polymerization mechanism (faster initiation and slower propagation and no termination process).

**Figure 7:**
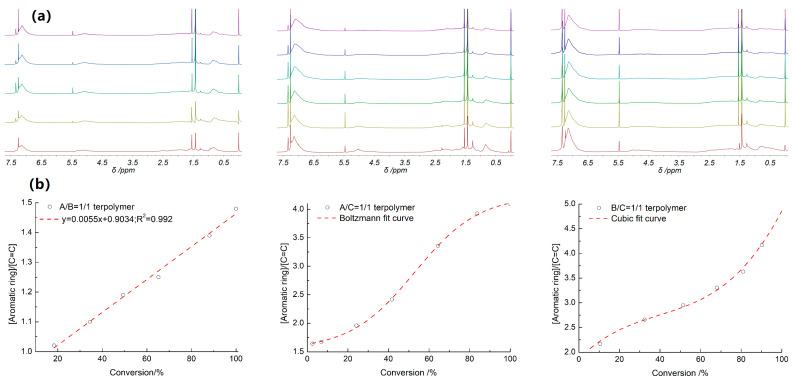
(**a**) ^1^H NMR spectrum of **A/B**, **A/C**, and **B/C** equivalent copolymerization in cyclohexane at 60 °C. (**b**) The instantaneous composition of the **AB/AC/BC** copolymer upon conversion.

**Scheme 3:**
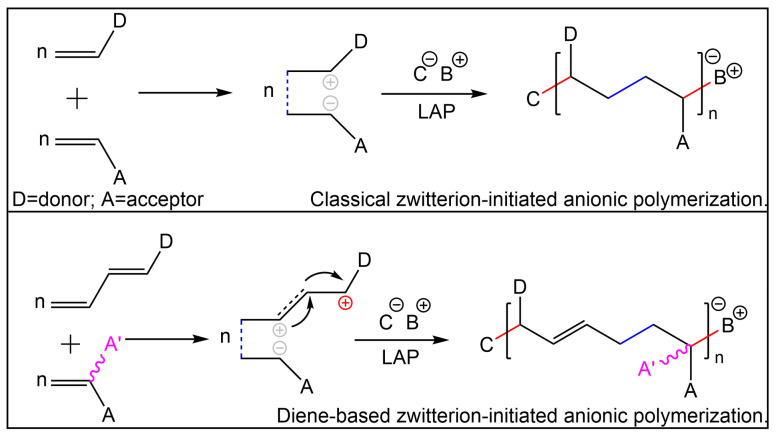
The illustration of zwitterion-initiated anionic polymerization [27].

Figure 7a shows the ^1^H NMR spectrum of several poly-(DPE-*co*-PD-*co*-St) terpolymers with 33.3 and 50 mol % of DPE, respectively. In addition to the chemical shift at 0.8 ppm corresponding to CH_3_ in 1,4-PD units and a band between 1.6 and 1.7 ppm corresponding to CH_3_ of 1,2-PD in the polymer backbone, there are two additional chemical shifts around 2.35 and 6.5–7.5 ppm, corresponding to Ph**-**C**H_2_-** (Ph-C**H**-Ph) aliphatic and aromatic protons in St and DPE units, respectively. As clearly shown in Figure 7, the mole ratio of [Aromatic ring]/[C=C] in the terpolymer was determined by the NMR technique, by comparing the integrated intensity between the chemical shift at 4.5–5.5 ppm, corresponding to the C=C group in the PD unit, and the absorption at 6.5–7.5 ppm, corresponding to the phenyl group in the St or DPE unit. In fact, a broad absorption at 6.0–6.5 ppm indicates the presence of SS microblock parts, which are clearly shown in **A/C** copolymerization cases. By constrast, the styrene homopolymerization reaction rarely occurred under the high feed ratio of DPE and never occurred in the case of **A/B** copolymerization. Figure 7b also shows the relationship of [Aromatic ring]/[C=C] in the terpolymer with the conversion determined by the corresponding ^1^H NMR spectra of terpolymers obtained at equivalent **A/B**, **A/C**, and **B/C** feedstock ratios, respectively. Overall, the anionic terpolymerizations show excellent activities attributed to the comparative reactivities of **A, B**, and **C** “monomers” and thus give excellent incorporation of DPE. To the best of our knowledge, such an effective incorporation of DPE is very difficult to achieve by traditional copolymerization systems, such as DPE/St [22]. Specifically, in the PD-rich case involving only **A** and **B**, the [Aromatic ring]/[C=C] increased linearly with the conversion of the comonomers, and the composition of the final poly (**A**-*co*-**B**) agreed well with the feedstock ratio ([Aromatic ring]/[C=C]~1.5). Following Hall’s theory of “tetramethylene zwitterion-initiated polymerization” [27], we deduced that the true initiating species is the tetramethylene zwitterion diradical formed by the reaction of two olefins of different polarities (Scheme 3). Both DPE and St (acceptor monomer) can form a zwitterionic tetramethylene intermediate when an excess of PD (donor monomer) is added to the system. As discussed in our previous paper, no consecutive St or DPE incorporation was observed in **A** or **B** ‘’homopolymerization’’ due to the stability of the zwitterionic intermediates. Moreover, the PD moiety had priority to interact with DPE (a stronger electron-deficient monomer) to form stable intermediate **B**, but the steric effect slightly decreased the copolymerization reactivity of **B**. Thus, the poly(**A**-*ran*-**B**) with gradient sequence distribution could be easily produced due to the similar reactivity ratios (*r***_A_** and *r*_B_). As for this unique case, the copolymer composition is basically determined by the monomer feed ratio and can also be easily controlled by the polymerization process. By contrast, the incorporation of the aromatic ring seemed to be quite insensitive to the feed ratio and monomer conversion, and the incorporation of PD is very constant at about 50 mol % as determined from the NMR data.

In general, the terpolymer composition was also in agreement with the comonomer ratio in the case of **A/C** copolymerization. Even at a high concentration of St, a small proportion of DPE still had a competitive reaction with the PD moiety to give **B** intermediates, which underwent anionic copolymerization to give an alternating sequence. The primary constant ratio of [Aromatic ring]/[C=C] in the terpolymer at *x* < 20% ranged from 1.50 to 1.75 (Figure 7), indicating the participation of **B** or St in the initial terpolymerization process. By contrast, the complete conversion of DPE and the formation of SSS microblock parts all proved this point. Therefore, all the St monomers cannot cross-propagate to give a strict sequence structure as **A** and **B** zwitterion forms. However, the residual St can still undergo homo-propagation to extend the macromolecular chain due to its living characteristics. On the contrary, DPE as the main monomer was expected to be inserted into the growing chain in the forms of the alternating sequence (**B** or **C**) and cannot be immediately and completely consumed within 3 h under our optimized conditions. Of course, on the one hand, the DPE exhibited good alternating activity in the copolymerization of PD but showed poor alternating activity toward the St monomer. The stability of zwitterion **B** was much higher than that of zwitterion **C** due to the poor electron-rich characteristic of St comonomer. On the other hand, nearly all St and PD monomers were completely consumed in the case of DPE-rich terpolymerization. The crossover propagation rate of St to DPE was slower than that of St to PD, and the St homopolymerization rate was impossible to ignore. Under ideal conditions, a perfect alternating copolymer of S and DPE, containing 63 wt % of DPE repeat units, could be obtained. It was well known that increasing the ratio of DPE to St would push the level of DPE incorporation toward the theoretical limit. However, the highest of the DPE incorporation ratio obtained in the **B/C** = 1/1 case was only about 90% ([Aromatic]/[C=C] = 4.5 at *x* = 91%). Significantly, the DPE incorporation determined by NMR increased up to 95% when the *f***_C_** was above 0.8 at 60 °C, where *f***_C_** is the mole fraction of the **C** “monomer” fed to the reactor. These results, combined with the inability of DPE to homopolymerize, contributed to the establishment of unique sequence distributions within each terpolymer. In addition, some evidence is shown in Figures S4–S7. The ^1^H NMR spectra of Poly (**A**-*co*-**B**)/Poly (**A**-*co*-**C**)/Poly (**B**-*co*-**C**) copolymers in CDCl_3_ are shown in Figure S4. The absence of peaks at *δ* = 5.5 ppm indicated the absence of the PD homopolymer sequence, and the low intensity of or even absence of absorption peaks at *δ* = 6.0~6.5 ppm also proved the random or alternating sequence distribution of St monomers. The ^13^C NMR spectra of Poly (**A**-*co*-**B**)/Poly (**A**)/Poly (**B**) copolymers are shown in Figure S5. It clearly shows that the alternating sequence structures of **A** and **B** are well preserved in Poly (**A**-*co*-**B**) [20,21]. Moreover, the three copolymers Poly(**A**-*co*-**B**)/Poly(**A**-*co*-**C**)/Poly(**B**-*co*-**C**) were compared and analyzed by ^13^C NMR spectra shown in Figures S6 and S7, and it showed that the **A/B/C** alternating sequences were relatively regular.

**Figure 8:**
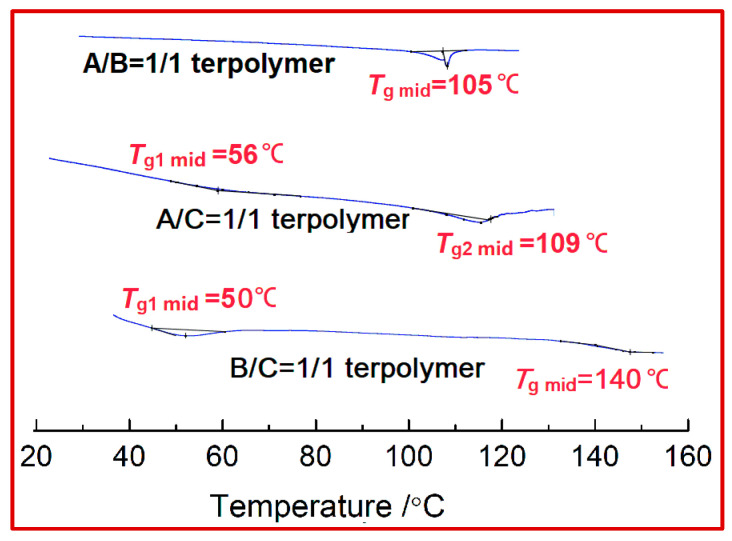
DSC spectra of equivalent **A/B**, **A/C**, and **B/C** terpolymers.

It was interesting to note that the composition and microstructure sequence distribution in the polymer chain determined the properties of the materials. The thermal properties of these terpolymers (*T*_g_s) were investigated by differential scanning calorimetry (DSC). Figure 8 shows several DSC curves of **A/B**, **A/C**, and **B/C** terpolymers (a 1/1 feed ratio at 60 °C). The **A/B** curve has only a sharp *T*_g_ transition in a flat baseline with no detectable melting point. The combination implies a homogeneous terpolymer microstructure with a completely amorphous morphology. The similar DSC curve was observed in the **A/C** sample shown, but an unnoticeable *T*_g_ (~56 °C) can be attributed to the presence of SSS microblock parts with low molecular weights. As for the DPE-rich terpolymers, the *T*_g_ is clearly found as a function of the DPE and St as well as PD content. In addition, the higher DPE incorporation enhanced the thermal property of this DPE-rich terpolymer (**B/C**) with a *T*_g2_ value up to 140 °C. Another glass temperature transition (*T*_g1_ ~50 °C) was derived from the **B**-rich “taper” block with low molecular weight. Overall, the exhibition of these DSC spectra was in accordance with the kinetic and NMR analysis.

**Scheme 4:**
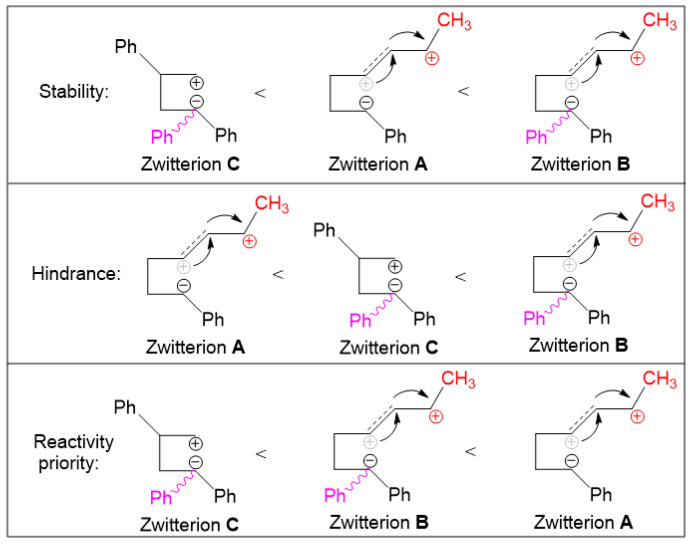
The stability, hindrance, and reactivity difference of **A/B/C** zwitterions in this work.

**Scheme 5:**
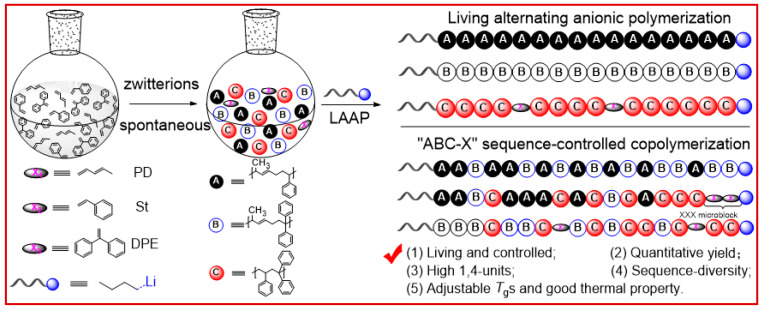
The proposed “**ABC-X**” sequence-controlled terpolymerization in this work.

As shown in Scheme 3, the tetramethylene intermediate may be predominantly zwitterionic in its electron distribution, depending on the terminal substituents of D/A comonomers. As for the 1,3-diene moiety with the electron-donating group (−CH_3_ in this work), it was reasonable to propose that the deformable tetramethylene can be easily initiated by anionic species in the case where the electron-rich (St and DPE) and electron-poor (PD) olefins are polymerizable. Considering the hindering effect of the terminal group in the acceptor monomer and the stability of the formed zwitterions, the reactivity ratio is listed in this order: zwitterion **A** > zwitterion **B** > zwitterion **C** (Scheme 4). According to the classical “bond-forming initiation” theory [27], our proposed “**ABC-X**” sequence-controlled terpolymerization mechanism was applied to interpret the polymerization behavior and the resulting novel sequences of these terpolymers, which is completely consistent with the reaction scheme shown in Scheme 5. To the best of our knowledge, although the initiation of cationic homopolymerization by a zwitterionic intermediate has been amply demonstrated, the anionic homopolymerization initiated by a zwitterionic intermediate is still seldom reported. Additionally, we believed that this is an interesting and rare example to be investigated in the field of anionic polymerization. Taking into account the advantages of living anionic polymerization, we are also very confident that many new, perfect, well-defined, and well-controlled sequences could be designed and synthesized with the help of this unique mechanism.

## 3. Conclusions

Herein, we have reported kinetic studies of the living sequence-controlled terpolymerization of PD with two styrene derivatives to afford polydiene-based resins with high molecular weights and very narrow molecular weight distributions using *n*-BuLi initiator in the presence of equivalent THF. The kinetic parameters for the process involving DPE/PD/St monomers in cyclohexane solution had a direct relationship with feed ratios and temperatures, where either of the two comonomers can undergo living anionic polymerization to afford a rigid and well-defined alternating copolymer under mild conditions. For the proposed zwitterionic intermediates **A/B/C**, the relative competitive activity decreased in the order **A** > **C** > **B**; however, quantitative terpolymers could be obtained in almost all **AB/AC/BC** terpolymer cases. Of course, the ^1^H NMR spectra tracking the instantaneous terpolymer composition with a yield in equivalent **AB/AC/BC** terpolymerization cases indicated these distinctive copolymerization behaviors. Furthermore, a linear 1,4-addition reaction occurred predominantly for the insertion of PD units in all terpolymerization cases. In this amorphous terpolymer product resulting from the copolymerization of **A** and **B**, only one *T*_g_ was observed at 105 °C, while two vastly different *T*_g_s were observed in the other two cases. We have proposed that the untypical *T*_g_ value to be the result of some microblock parts of free monomer (**X**) homopolymerization as well as the competition reaction (transformation of partial zwitterion **B** to **C**). The “alternating module”-free monomer (**ABC-X**) mechanism was proposed to illustrate the terpolymerization process, which belongs to the classical “bond-formation initiation” theory in anionic polymerization.

## Figures and Tables

**Table 1:** Living anionic copolymerization of **A**[St¦PD]/**B**[DPE¦PD]/**C**[St¦DPE] with a different feed ratio using *n*-BuLi as an initiator in cyclohexane.

Comonomers ^(a)^		Feed Ratio	T	Time	Yield	*M*_n_ ^(b)^	*Ð* ^(b)^
M_1_	M_2_	n_1_/n_2_	[°C]	[min]	[%]	[kg/mol]	
**A**	**B**	02:08	70	120	>99	22.3	1.12
**A**	**B**	04:06	70	120	>99	31.9	1.09
**A**	**B**	05:05	70	60	>99	25.5	1.08
**A**	**B**	06:04	70	60	>99	26.4	1.09
**A**	**B**	07:03	70	60	>99	24.6	1.08
**A**	**B**	08:02	70	60	>99	30	1.07
**A**	**B**	09:01	70	60	>99	21.9	1.09
**A**	**C**	03:07	70	60	>99	20.9	1.06
**A**	**C**	05:05	70	40	>99	19.9	1.09
**A**	**C**	06:04	70	40	>99	25.1	1.07
**A**	**C**	07:03	70	40	>99	23.5	1.07
**A**	**C**	08:02	70	40	>99	19.4	1.1
**A**	**C**	09:01	60	120	>99	49.4	1.13
**B**	**C**	02:08	60	120	>99	23.4	1.06
**B**	**C**	03:07	60	60	90.5	12.7	1.09
**B**	**C**	05:05	50	180	91.2	41.6	1.13
**B**	**C**	06:04	60	120	90.8	28.4	1.07
**B**	**C**	07:03	50	180	91.6	36.6	1.13
**B**	**C**	08:02	70	180	>98	40.2	1.1

^(a)^ All polymerization reactions were performed in cyclohexane with *n*-BuLi as initiator: monomer/solvent = 0.3 g/mL, [*n*-BuLi]_0_ = 1~2 × 10 ^−5^ mol·L^−1^. ^(b)^ Determined by gel permeation chromatography (GPC), all original curves are shown in Figures S1–S3 (Supporting Information).

## Data Availability

Not applicable.

## Supplementary Materials

The following supporting information can be downloaded at: https://www.mdpi.com/article/10.3390/polym15092191/s1, Figure S1: GPC of A/B copolymers with different feed ratios; Figure S2: GPC of A/C copolymers with different feed ratios; Figure S3: GPC of B/C copolymers with different feed ratios; Figure S4: 1H NMR spectra of Poly (A-co-B)/Poly (A-co-C)/Poly (B-co-C) copolymers in CDCl3; Figure S5: 13C NMR spectra of Poly (A-co-B)/Poly (A)/Poly (B) copolymers in CDCl3; Figure S6: 13C NMR spectra of Poly (A-co-B)/Poly (A-co-C)/Poly (B-co-C) copolymers in CDCl3 (132 ppm < δ < 152 ppm); Figure S7: 13C NMR spectra of Poly (A-co-B)/Poly (A-co-C)/Poly (B-co-C) copolymers in CDCl3 (124 ppm < δ < 131 ppm). Reference [28] is cited in the supplementary materials.
