# Supplementary material for: 1,3-Pentadiene-Assistant Living Anionic Terpolymerization: Composition Impact on Kinetics and Microstructure Sequence Primary Analysis
Source: Polymers (Basel). 2023 May 5;15(9):2191. doi: 10.3390/polym15092191 (PMC10180649; doi:10.3390/polym15092191)
Supplement: Supplementary file 1 [file polymers-15-02191-s001.zip › polymers-2336149-supplementary.pdf]

polymers

Supplementary Information

1,3-Pentadiene-Assistant Living Anionic Terpolymerization: Composition Impact on Kinetics and Microstructure Sequence Primary Analysis

Qiaoqiao Xiong [1,†], Yawen Fu [1,†], Jundong Xu [1,†], Zhuowei Gu [1], Chengjun Peng [1], Haoyun Tan [1], Qiqi Dai [1], Yujie Cao [1], Fengli Xie [1], An Li [1], Wenjun Yi [2], Lijun Li [1,*] and Kun Liu [1,*]

[1] Province Key Laboratory for Fine Petrochemical Catalysis and Separation, College of Chemistry and Chemical Engineering, Hunan Institute of Science and Technology, Yueyang 414006, China

[2] College of Materials Science and Engineering, Changsha University of Science &Technology, Changsha 410082, China

* Correspondence: lilijun115@163.com (L.L.) liukun328@126.com (K.L.)

† These authors contributed equally to this work.

Experimental

Materials. All manipulations were performed under an argon atmosphere using a Mikrouna glove box. All reagents were purchased from Aldrich unless otherwise noted. Tetrahydrofuran (THF) and cyclohexane were distilled from sodium/benzophenone under argon and then thoroughly degassed before use. 1,1-Diphenylethylene (DPE) and styrene were distilled over a small amount of CaH_2 with stirring for 24 h and distilled under high vacuum prior to use. *n*-BuLi (2.4 mol·L^{-1} in *n*-hexane) was used without any further purification. Anhydrous methanol was sparged with argon for 15 min to remove dissolved oxygen and dried by activated molecular sieves (4Å). 1,3-Pentadiene (polymer grade) was purified according to the literature procedures [1].

Measurements. The number-average molecular weights (M_n) and molecular weight distributions ($Đ_M$) of the polymer samples were determined using a Waters GPC liquid chromatograph (Waters, Milford, USA) equipped with gel columns (300 × 7.8 mm). THF was used as eluent, and the flow rate was 1.0 mL·min^{-1} at *r.t.* A molecular weight calibration was established using polystyrene (PS) standards.

The ^1H NMR spectra of the polymer samples were recorded on a Varian INOVA-400 spectrometer(Varian, Palo Alto, USA) at room temperature. Chemical shifts were recorded in ppm downfield relative to $CDCl_3$ (δ = 7.26 ppm) for ^1H NMR as a standard. The T_g values of the polymer samples were measured by DSC using a NETZSCH instrument DSC200F3 apparatus(NETZSCH, Selb, Germany) under nitrogen. The polymer samples were first heated to 150 °C, cooled to 20 °C, and then scanned at a rate of 10 °C·min^{-1}.

Typical Procedure for 1,3-Pentadiene (PD) Homopolymerization. A detailed polymerization procedure is described as a typical example. In the glove box, *n*-BuLi initiator (15 μmol) was first dropped into 1 mL of dry and degassed cyclohexane. Then 1 equiv of THF (15 μmol) was added with stirring to give a pale yellow or colorless solution. After a few minutes, 0.30 g of PD (4.4 μmol) was finally added and the reaction was carried out at 80 ° C for 8 hours to obtain a viscous solution. The reaction was then terminated by adding a small amount of acidic methanol containing 2,6-di-tertbutyl-*p*-cresol (1 wt %) as an antioxidant reagent. The resulting polymer was poured into a large quantity of methanol and then dried under vacuum at room temperature to a constant weight (0.30 g, 100%).

Typical Procedure for Copolymerization of 1,3-Penadiene and St or DPE. A typical copolymerization reaction is shown below. In a glove box, a hexane solution (0.1 mL) of *n*-BuLi (15 μmol) was added to a cyclohexane solution (1 mL) in a 10 mL Schlenk tube.

The mixture was stirred at *r.t.* for a few minutes, and then the mixture of 0.181 g (1.74 mmol) of St and 0.119 g (1.74 mmol) of PD, or the mixture of 0.218 g (1.20 mmol) of DPE and 0.082 g (1.20 mmol) of PD was added under vigorous stirring. After 2 hours, the reaction was terminated by adding a small amount of acidic methanol containing 2,6-di-tert-butyl-*p*-cresol (1 wt %) as an antioxidant reagent. The mixture was poured into methanol (20 mL) to precipitate the copolymer product. The copolymer was collected by filtration, and dried under vacuum at 30 ° C to a constant weight (0.30 g, 100%).

Typical Procedure for Tercopolymerization of Styrene with DPE and PD. A typical terpolymerization reaction is shown below (Table 2, run 4). In the glove box, an *n*-hexane solution of *n*-BuLi (150μL, 15 μmol) was added to a cyclohexane solvent (1 mL) in a 10 mL flask. The mixture was stirred at 20 ° C for a few minutes, and the mixture of 0.096 g (0.922 mmol) of styrene and 0.063 g (0.922 mmol) of PD and 0.166 g (0.922 mmol) of DPE was added under vigorous stirring. After 20 min, the reaction was terminated by adding a small amount of acidic methanol containing 2,6-di-tert-butyl-p-cresol (1 wt %) as an antioxidant reagent. The mixture was poured into methanol (20 mL) to precipitate the terpolymer product. The terpolymer was collected by filtration, and dried under vacuum at 40 ° C to a constant weight (0.30 g, 100%).

SI Figures

Figure S1 GPC of **A/B** copolymers with different feed ratios.

Figure S2 GPC of **A/C** copolymers with different feed ratios.

Figure S3 GPC of **B/C** copolymers with different feed ratios.

Figure S4 ^{1}H NMR spectra of Poly **(A-*co*-B)**/Poly **(A-*co*-C)**/Poly **(B-*co*-C)** copolymers in $CDCl_3$.

Figure S5 ^{13}C NMR spectra of Poly **(A-*co*-B)**/Poly **(A)**/Poly **(B)** copolymers in $CDCl_3$.

Figure S6 ^{13}C NMR spectra of Poly **(A-*co*-B)**/Poly **(A-*co*-C)**/Poly **(B-*co*-C)** copolymers in $CDCl_3$ (132 ppm < δ < 152 ppm).

Figure S7 ^{13}C NMR spectra of Poly **(A-*co*-B)**/Poly **(A-*co*-C)**/Poly **(B-*co*-C)** copolymers in $CDCl_3$ (124 ppm < δ < 131 ppm).

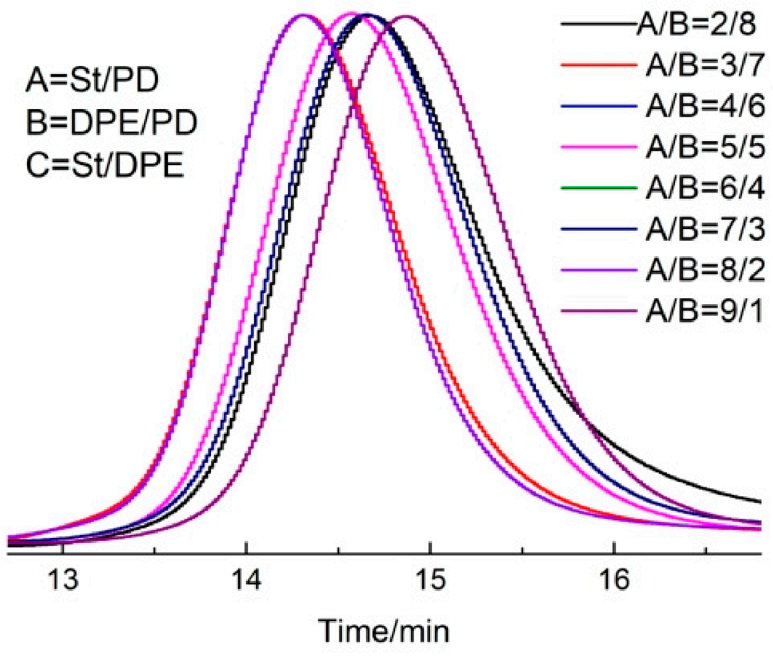

Figure S1. GPC of **A/B** copolymers with different feed ratios.

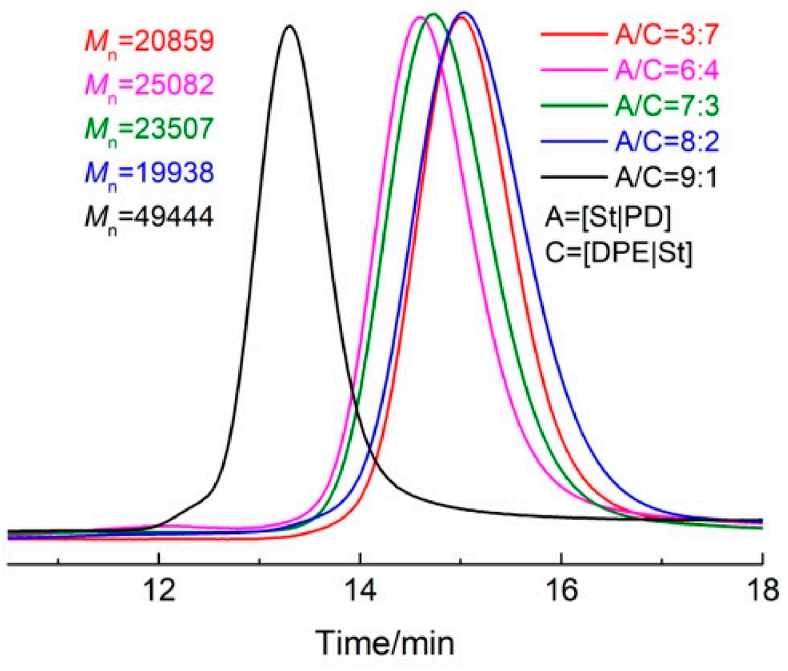

Figure S2. GPC of **A/C** copolymers with different feed ratios.

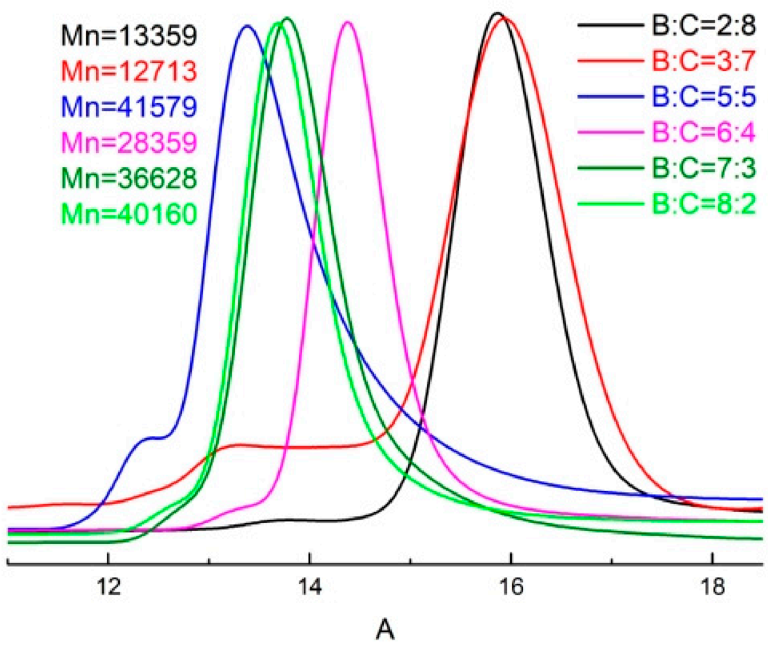

Figure S3. GPC of **B/C** copolymers with different feed ratios.

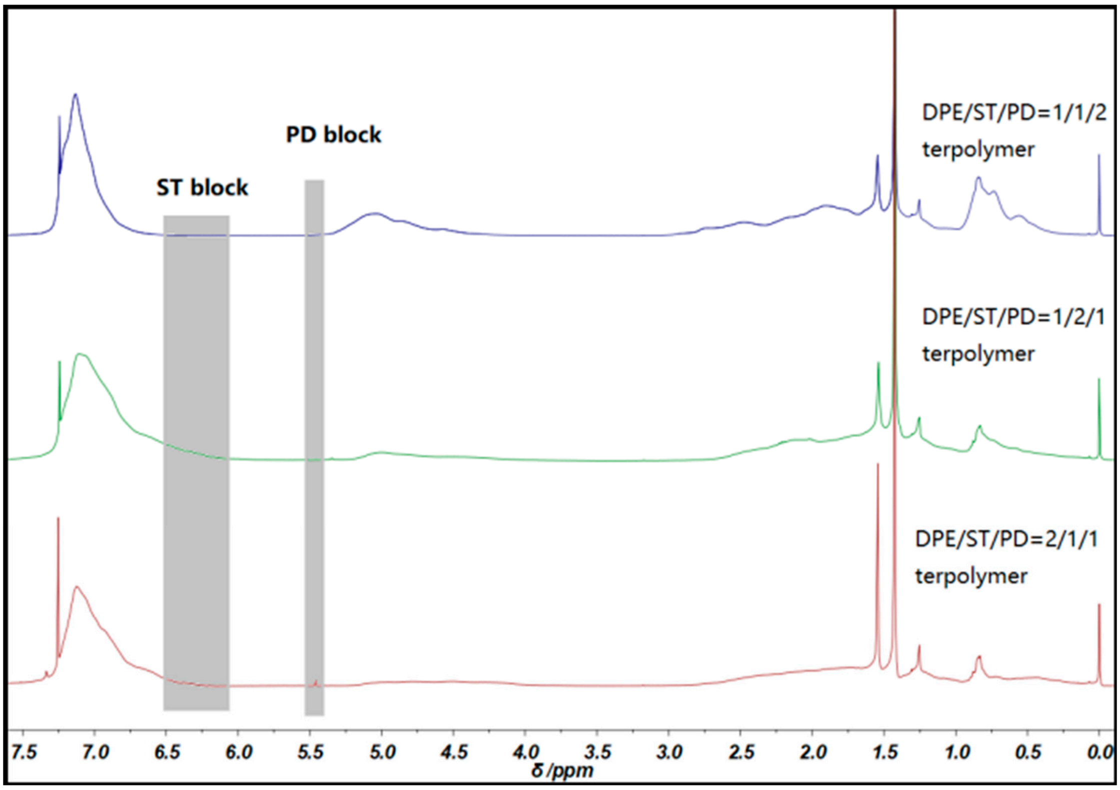

Figure S4. ^{1}H NMR spectra of Poly **(A-*co*-B)**/Poly **(A-*co*-C)**/Poly **(B-*co*-C)** copolymers in $CDCl_3$.

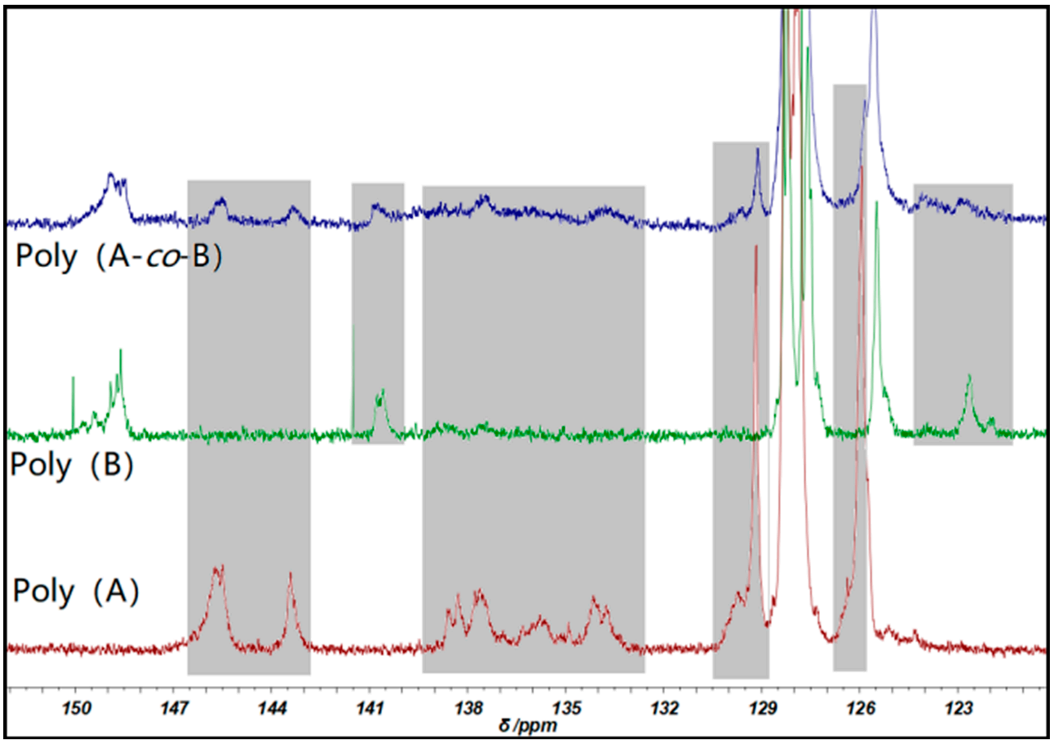

Figure S5. ^{13}C NMR spectra of Poly **(A-*co*-B)**/Poly **(A)**/Poly **(B)** copolymers in $CDCl_3$.

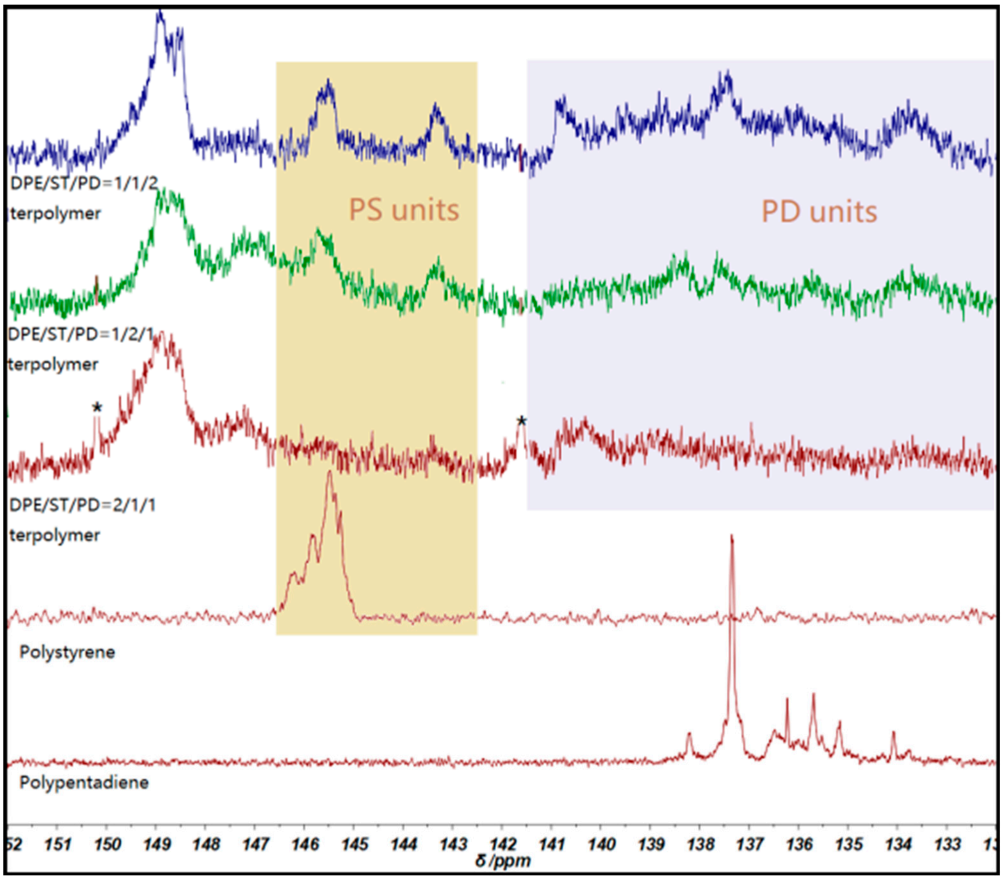

Figure S6. ^{13}C NMR spectra of Poly **(A-*co*-B)**/Poly **(A-*co*-C)**/Poly **(B-*co*-C)** copolymers in $CDCl_3$ (132 ppm < δ < 152 ppm).

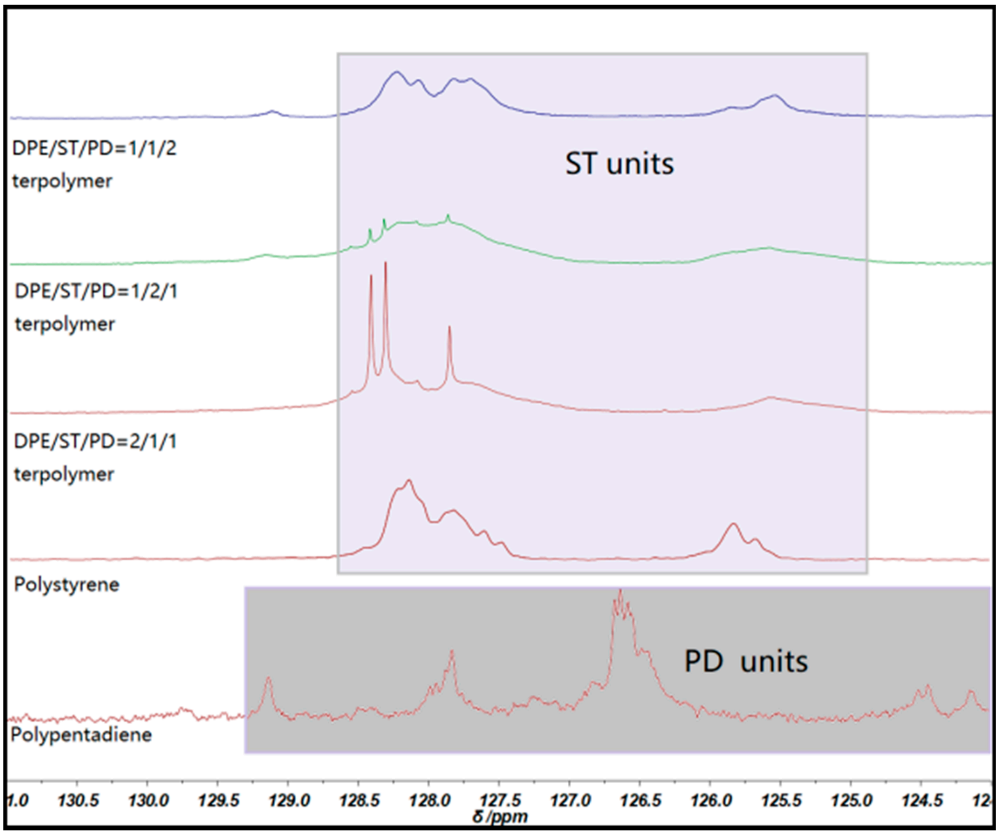

Figure S7. ^{13}C NMR spectra of Poly **(A-*co*-B)**/Poly **(A-*co*-C)**/Poly **(B-*co*-C)** copolymers in $CDCl_3$ (124 ppm < δ < 131 ppm).

Reference

[1] Liu K, He Q, Lei R, Feng X, Wei J X Living anionic polymerization of (E)-1,3-pentadiene and (Z)-1,3-pentadiene isomers. *Journal of Polymer Science Part A Polymer Chemistry* **2016**, 54(15): 2291-2301.
